# From the Public Health Perspective: a Scalable Model for Improving Epidemiological Testing Efficacy in Low- and Middle-Income Areas

**DOI:** 10.2196/55194

**Published:** 2024-06-10

**Authors:** Xuefeng Huang, Qian-Yi Kong, Xiaowen Wan, Yating Huang, Rongrong Wang, Xiaoxue Wang, Yingying Li, Yuqing Wu, Chongyuan Guan, Junyang Wang, Yuanyuan Zhang

**Affiliations:** 1 School of Public Health Dalian Medical University Dalian China; 2 School of Economics and Management University of Science and Technology Beijing Peking China; 3 School of Economics and Management Jiangxi University of Traditional Chinese Medicine Nanchang China

**Keywords:** low- and middle-income country, LMIC, pandemic, epidemiological surveillance, universal public health, nonpharmacological interventions, public health, callable model, efficacy, COVID-19, public safety threats, effectiveness, China, detection, epidemic

## Abstract

The globe is an organically linked whole, and in the pandemic era, COVID-19 has brought heavy public safety threats and economic costs to humanity as almost all countries began to pay more attention to taking steps to minimize the risk of harm to society from sudden-onset diseases. It is worth noting that in some low- and middle-income areas, where the environment for epidemic detection is complex, the causative and comorbid factors are numerous, and where public health resources are scarce. It is often more difficult than in other areas to obtain timely and effective detection and control in the event of widespread virus transmission, which, in turn, is a constant threat to local and global public health security. Pandemics are preventable through effective disease surveillance systems, with nonpharmacological interventions (NPIs) as the mainstay of the control system, effectively controlling the spread of epidemics and preventing larger outbreaks. However, current state-of-the-art NPIs are not applicable in low- and middle-income areas and tend to be decentralized and costly. Based on a 3-year case study of SARS-CoV-2 preventive detection in low-income areas in south-central China, we explored a strategic model for enhancing disease detection efficacy in low- and middle-income areas. For the first time, we propose an integrated and comprehensive approach that covers structural, social, and personal strategies to optimize the epidemic surveillance system in low- and middle-income areas. This model can improve the local epidemic detection efficiency, ensure the health care needs of more people, reduce the public health costs in low- and middle-income areas in a coordinated manner, and ensure and strengthen local public health security sustainably.

## Introduction

Globally, advances in epidemiological, behavioral, and pharmacological interventions have led to a decline in the incidence of sudden public health events. However, in some low- and middle-income countries (LMICs), large-scale outbreaks of various communicable or noncommunicable diseases have not been proportionately contained, and the burden on public health continues to rise [1].

Current interventions for epidemiological prevention are suboptimal and tend to be high-cost and decentralized. A typical recent manifestation of this is the fact that in the worldwide discussion of how to win the COVID-19 war, the rush to action has varied greatly from country to country, and even within countries. The US government advises against parties of more than 10 people, but San Francisco, California, has ordered everyone to stay home. Italy, France, and Spain have sealed off their populations almost completely, with police or military patrolling the streets in some places. Even as major cutting-edge magazines continue to report on the health threat of COVID-19, pubs remain open in Britain, Germany’s schools close, and Sweden’s schools remain open to young children. The patchwork reflects the different stages of the epidemic, as well as differences in resources, cultures, governments, and laws [2]. However, there has long been controversy about what works best, and how to balance the necessary with the reasonable, which some of the strategies adopted by these countries are missing elsewhere: widespread testing to detect cases, mass tracking of their contacts to test or quarantine them, and encouraging or forcing infected people to quarantine themselves. It is discrete and not cost-effective. Conversely, there is a large gradient in the timing of the blockades as income statuses change, with LMICs initiating disincentives earlier than ultra–high-income or high-income countries [3]. In the case of India, one of the first countries to observe the health impacts of neocoronaviruses and to adopt a containment strategy, the establishment of a comprehensive blockade strategy of varying degrees of severity was declared in 2020, but in April 2021, the second wave of the COVID-19 pandemic in the country inexorably shifted from the west and north to the east and south [4]. A study investigating the scale of the infection showed an increase in seroprevalence in India from just over 20% in January 2021 to 67% in June-July 2021 [5]. Initially, local scientists attributed the increase to a variant of the virus, but after several months of comparative studies, it was found that the variant was not more transmissible [6]. Although Indian public health researchers have stressed the need for continued vigilance and standardized genomic surveillance, there are no restrictions on major events in key states, especially elections or religious gatherings, where returnee positivity rates are very high. This has upset the balance of prevention systems in different states and cities, and the interaction of old and new problems has created huge economic gaps in the health systems of different regions. The concomitant shortage of resources due to drug abuse and declining drug resistance, as well as more cases of multiple comorbidities, seems to be a common problem in many low- and middle-income areas [7]. Another issue of concern is vaccination. Most countries use electronic registries, and despite the principle of harmonization, vaccination during pandemics has long been limited by availability. Moreover, unlike the excellent tracking of vaccination in high-income regions, there has been a major gap in the ability of low- and middle-income regions to estimate the magnitude of morbidity and mortality during pandemics accurately. Past mortality analyses suggest that socioeconomic deprivation during pandemics leads to more excess deaths [8], and this is only a preliminary assessment from regions with stronger health and data systems; some transitional regions or poorly informationalized tribes may be worse off than realistically predicted. The pandemic in India and the current state of vaccine distribution worldwide point the way to the future of pandemic prevention in LMICs—early signal detection, systematic identification and analysis of health systems and their supply chain preparedness, and protection of the ability to deal with not only emergencies but also other disease-care services, as well as the ability to collect, collate, integrate, analyze, and interpret in real time. The ability for data to be recycled so that resources can be appropriately allocated when needed and health systems can better aid in understanding the causes of both poor and favorable outcomes. In 2022, A research survey further reiterated that epidemiological surveillance tends to prioritize urban and adjacent rural communities, but it simply does not reach low-income or remote rural areas [9]. To address the current gaps in pre-epidemic surveillance and prevention, Mobarak et al [10] suggest creating a relatively complete and locally relevant general detection system, including individual and population-wide engagement strategies, in areas where the basic public health system lags relatively behind that of other higher-income areas, which would address some of the cost commonalities and resolve some social conflicts.

Nonpharmacological interventions (NPIs), as the most important part of the current epidemiological prevention and control system, are aimed at raising awareness of risk reduction activities at the individual level (eg, proactive wearing of face masks and promotion of social distancing, among others) and the group level through a range of public health strategies, as well as common actions assumed in response to public health outbreaks (eg, group testing and quarantine, among others). This decelerates the spread of disease and prevents larger outbreaks to ensure that hospitalization and mortality rates are manageable [11].

In fact, before the SARS-CoV-2 outbreak, many countries had made steady progress toward achieving Sustainable Development Goal target 3.4 (reducing premature mortality from noncontagious diseases by one-third between 2015 and 2030) [12]. Of note, influenza pandemics impede these successes, disrupt health systems, divert resources, and draw attention away from them. Therefore, an important fact about COVID-19 is that anticipating and being alert to viral risks in advance and building a strong public health system with supervision, detection, and timely responses is essential and will greatly benefit the rational allocation of resources to the district health system and provide it with a path for the future. However, in designing and operating a surveillance intervention system, consideration needs to be given to the interrelated structural factors that support or impede preventive actions and behaviors of individuals or groups. These structural factors can be variable or inherent to social patterns, such as socioeconomic and cultural conditions, political will (eg, cooperation, commitment, and funding), governmental priorities or policies, and conditions related to environmental factors (eg, reducing industrial pollution). Addressing these structural factors can improve the preventive effectiveness of public health and protect the health interests of more people, thereby achieving sustainable economic and health benefits [13].

## The Significance of Structured Disease Detection Systems in Low- and Middle-Income Areas

About half of the people in LMICs live in rural or remote areas [14]. Livestock raised in remote rural areas accounts for about 25% of the agricultural gross domestic product of LMICs and supports the livelihoods of more than 600 million smallholder farmers [15]. Complex environmental pathogenicity and high prevalence of zoonotic factors are a major problem for not only local but also global public health. In fact, previous pandemics have fueled a revolution in global disease prevention, including one in LMICs. It is emerging as a global consensus that vaccinating populations in remote areas will lead to a reduction in the risk of new viral mutations worldwide [16]. In many countries, domestic distribution capacity can be established quickly, affordably, and cost-effectively. LMICs have accumulated extensive experience with mass immunization campaigns, which have resulted in high levels of vaccine coverage for children and older adults [17]. Several countries, including Ghana, Liberia, India, Pakistan, and Sierra Leone, have begun experimenting with the concept of “mobile vaccination teams,” which bring batches of vaccines to the places where people live to make vaccinations more accessible [18,19]. These can involve nurses, supported by community mobilizers, visiting remote villages with vaccines to raise the attention of local residents and leaders, and calling for effective vaccination. This model has also been used successfully in the past: the “outreach clinics” in the Gambia, which provide immunization services to difficult-to-reach subpopulations (eg, homeless individuals, stigmatized groups, and migrant groups, among others), are considered an important factor in the elimination of measles [20]. This is mutually beneficial to the systematic monitoring scalable model we proposed. An efficient disease detection system can provide precise regional control and tools for local public health management, more effectively decelerate the spread of diseases in complex pathogenic environments, alleviate social conflicts in NPIs, enhance the vitality of public health, and provide a more precise guarantee of public health security and economic development in low- and middle-income areas. However, policy decisions are usually time- and resource-intensive and require a multidisciplinary team to implement. While policy support mechanisms tend to be effective in high-income countries, they are often inadequately monitored in many LMICs. Inadequate monitoring is largely due to budgetary constraints, which limit the ability to plan and coordinate. The use of affordable digital technologies can facilitate real-time monitoring and feedback from individuals, service providers, and policy makers. On the other hand, the theory of “synergy” has always played an important role in social science research and is often combined with governance theory by management scholars to create a more effective theory of “collaborative governance.” Thus, to address structural and other barriers to disease prevention and control, there is a need for a timely and more flexible, public, and dynamic system in place, which emphasizes the use of the total of the many ways in which individuals and various public or private institutions manage their commonalities [21], and which promotes public policy support mechanisms to guide policy decision-making in disease prevention.

## What Should We Do to Achieve Efficient Detection?

### Overview

**Figure 1:**
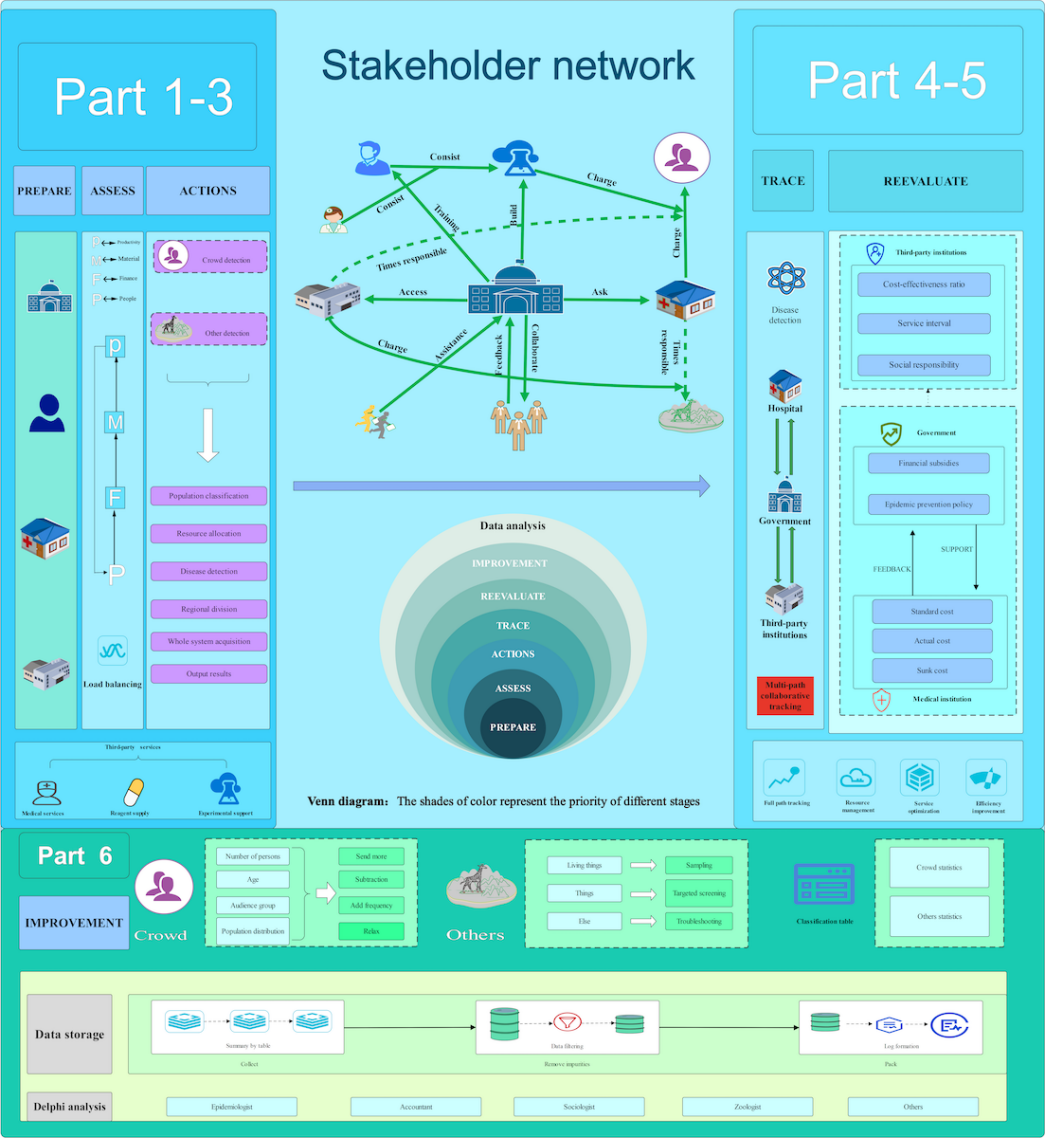
Model diagram for strengthening the effectiveness of epidemic detection in low- and middle-income areas, which would facilitate integration into existing surveillance systems.

As we discussed earlier, there has long been controversy about what works best and how to balance necessity with rationality. A complex pathogenic environment remains, but the past history of overscaled, short-lived surge operations has been unsystematic and wasteful. It brings not only lower–middle-income areas economic disasters during the pandemic but also the same or even greater vulnerability of local public health systems to a new round of public health challenges. So, we have transferred and quantified the traditional thinking on disease prevention and control to develop a disease prevention–sharing model with the government as the protagonist and the participation of the population, the health care system, and the private sector, and we have provided a service architecture diagram about epidemiological surveillance in low- and middle-income areas (Figure 1). Adopting the 4 dimensions of human, financial, material, and labor as the object of assessment and apportionment, this or the next public health detection event comprises 6 recyclable phases, including preparation, assessment, action, tracking, reassessment, and improvement, as well as an additional phase, with the first 6 phases operating in a closed loop. The efficacy assessment of each of these stages is based on our analysis of epidemiological data from selected low- and middle-income areas of south-central China, a comprehensive and targeted intervention design that is effective in the management of infectious disease illnesses such as pneumococcal pneumonia. Furthermore, this approach also can be considered for the management of noncommunicable or other epidemics such as zoonotic diseases.

### Phase 1: Every Part Is Ready

The primary aspect of improving disease detection efficacy in low- and middle-income areas is to strengthen the response mechanisms of various stakeholders and build a bundle with efficient linkages. Throughout the COVID-19 pandemic, everyone has been able to be a transmitter and everyone has been able to be a defender. In the face of the public enemy of humanity, all controversies and prejudices were temporarily set aside [22]. We have divided all participants into 4 categories: the first is the government or local governing body, the second is the public, the third is the medical organizations, and the fourth is the private organizations. Generally speaking, the connection between regional governments and regional medical institutions is very close, and most public health organizations always respond to the first call of regional governments to solve public health matters; private institutions also bear certain social responsibilities. Simultaneously, in their daily health business dealings, they cooperate with public medical units to lay a large stage of the contact mechanism as the preparation stage of the second response echelon. The response of the population is the slowest and most important presence in the whole detection system. The development of new media communication has greatly saved the time and material costs of health information dissemination, but given that a large portion of the population lives in remote rural areas, there is still a gap in the ability of these populations to perceive the message due to the variability of communication networks. To solve such problems, the Chinese government has set up small government-like management teams in each village, which are elected by the residents themselves. They tend to have higher education levels and political literacy, and a high degree of familiarity with the environment in which they are located, and the residents’ authority over them is even higher than that of other expatriates, so they can be more accurate and effective in delivering information and preventing risks in their localities. In addition, and more importantly, it is often much less costly to train them than it is to reach the general population. We may wish to use such a mechanism to achieve a more efficient detection and cost-saving pathway, combined with the effectiveness of new media communications to form a third echelon of the crowd response during the preparedness phase.

Thus, we can have a more linear public health emergency response pathway, and when the government issues instructions for the preparedness phase, the first and second response echelons can have a clearer preparation plan for material preparation and personnel control according to the different levels of demand of the third response echelon, which provides an introductory for later detection effectiveness tracking and cost analysis.

### Phase 2: Load Balancing of All Available Resources

Recommendations have been offered for nongovernmental organizations, governments, donors, and future research including studying the organizational effectiveness and sustainability of these partnerships to deliver effective and efficient health outcomes to recommend universal best practices in health care [23]. In disease detection systems in low- and middle-income areas, the usage and translational benefits of limited public health resources have a direct impact on the continuation of detection efforts. Load balancing is a quantitative assessment and optimization of resource interchange based on all available human, financial, material, and physical resources that the local area has. The 3-level response echelon should combine the actual local public health resources with the epidemic burden, analyze the public health cost of safeguarding the most realistic population’s basic health care needs at the emergent stage, and then perform resource interchange. Specifically, we start from 4 dimensions, including human, financial, material, and productivity dimensions, based on off-epidemic control, and the load capacity of each dimension in different regions needs to be precise to unit order of magnitude. For example, the number of people served by medical centers at all levels, the number of local funds invested in public health, the maximum number of testing reagents that can be provided, the maximum number of patients that can be admitted locally, etc, to complement each other’s strengths, visually help make up for the public health shortcomings of different regions in terms of funds or talents, optimize short-term benefits, and continuously mobilize other potential resources to maintain a more regular and sustainable development strategy.

In the previous phase of preparation, we emphasized the importance of response mechanisms, where the participation of all stakeholders enhances the carrying capacity of regional pre–disease detection efforts. The quantification of load balancing can effectively stimulate the participation of stakeholders to meet the simplest health rights needs of the widest local population in the pandemic era. On the one hand, it is an effective way to stimulate the work capacity of government and other health personnel, and on the other hand, it is a way to give direction and assistance to other stakeholders who are willing to take more social responsibility, thus maximizing the potential local public health capacity. The government as the core of the work can establish a public mechanism after quantifying the load capacity, and a joint class of government personnel and media to disclose the progress and cost budget of disease detection and prevention and control work at each stage to the population so that the transformation among human, financial, material, and productivity can have more flexible possibilities due to the participation of more aspects.

### Phase 3: Detecting General Differences in Actions May Be the Key to Cost Savings

The collaboration of stakeholders who have gone through the first 2 phases provides a clear framework and a more convenient environment for our next testing initiatives. We also need to take into account the general differences in population and environment when conducting disease detection in low- and middle-income areas. On the one hand, most people in low- and middle-income areas live in rural or remote areas and depend on the agricultural economy for their livelihoods, with low external mobility and relatively high levels of self-sufficiency. It is worth noting that these groups tend to have far more frequent contact with the natural environment (the novel coronavirus was originally identified as being carried by bats [24]). On the other hand, the degree of aging has always been at a high level in the rural population, so even though in the first phase of our preparation of the links to improve the attitude of the population, especially among older groups to cooperate and respond to the current disease, most of them are only motivated by the importance of their health care, while other disease-causing factors around them, such as food, domestic animals, work environment, etc, have a lower deficit of prevention awareness. General differences in population characteristics and the complexity of disease environments that are difficult to monitor have been important reasons underlying the difficulty of timely and effective control of outbreaks in low- and middle-income areas, and even higher disease mortality rates. Such a situation requires us to prioritize environmental and “special population” factors in the detection system, and governments, public health organizations, and other private institutions need to adopt a more appropriate multifaceted cooperation mechanism to achieve population and environmental biases of resources, effectively and precisely cut off the transmission of diseases, protect special populations, and thus save long-term testing costs.

Based on this, to achieve the efficiency and sustainability of the testing initiative, we suggest that the government could take the lead in responding to the third-tier government-like personnel and public health organizations in cooperation with private institutions to establish a special disease testing service site in each tribe of the low- and middle-income areas by measuring the geographic population and demand differences. The site could be staffed by local government workers with village doctors, and the testing laboratory and testing reagents and other equipment could be outsourced by a third-party organization. Adhering to the purpose of prioritizing the health testing needs of special populations and maintaining the living environment, we can choose to prioritize the training of these government workers and village doctors on disease prevention knowledge about collecting the disease testing needs of local special populations and the environmental risks of suspected transmission routes that need to be urgently cut off, so that when risk factors are found in the NPI work process, they can be fed back promptly to the service station. The medical staff of the service station can then directly implement testing and intervention, thus changing the local passive testing prevention system.

### Phase 4: All Traces of Traced

The main reason why, in the previous phase, each task needs to be divided so carefully is to achieve full pathway effectiveness tracking in this phase, thus reducing internal suspicion to a greater extent and achieving a relatively stable phase cycle. In low- and middle-income areas, a series of problems such as unprofessional medical personnel, unclear division of functions between government and other agencies, and simplistic and one-size-fits-all disease prevention processes have been running through the whole stage of disease outbreaks or suboutbreaks. This is resulting in the emergence and spread of sudden epidemics in low- and middle-income areas, once they are prone to difficult preventive measures and slow effectiveness of control means, and such recurrence has led many low- and middle-income regional governments to find it difficult to cope with the cost overlap, and this even causes them to choose to adopt the sluggish strategy of abandoning detection and relaxing preventive control.

### Phase 5: Another Small Revolution in Costs

At this stage, we have a general framework for disease detection, and some cost-trimming is required. The government, as the main body of action, needs to, on the one hand, give new load ratios based on the standard costs, actual costs, and sunk costs of the testing process as fed back by the hospitals in conjunction with the initial assessment, and then develop appropriate financial subsidy policies and epidemic prevention regulations to adjust the targets and outcome outputs of the testing work of medical institutions. On the other hand, it is necessary to promptly assess the cost-effectiveness of the testing services provided by third-party institutions throughout the testing service phase and determine the effect of social responsibility, to delineate new testing service intervals.

### Phase 6: Time to Make Some Changes

The first 5 phases provide the basis for creating meaningful improvements in the improvement phase. During the outbreak period, we have had the experience of the first round, the population detection strategy that can be adjusted to areas with high susceptibility or population mobility. With limited detectors, a deployment system that can be adapted to assign additional personnel to besiege high-incidence areas, the ability to abate the detection force of the extensive population, and prevent and control with precision, the government can redivide the areas and classify them to guide the medical institutions’ service bias. The work of environmental testing can also be based on the susceptibility of different objects to emphasize the transformation of responsibilities between third-party testing agencies and public health agencies, targeting biological testing with a higher effectiveness bias than nonbiological testing to prevent the emergence of new forms of transmission such as zoonotic diseases that cause more serious economic losses. In the small outbreak period or stabilization period, all aspects of the detection force need to return to the 3 stages of tracking link posts, narrowing the envelope, strengthening the regularity of detection laws, reducing the frequency, shrinking the detection object at the same time, according to the cost criteria assessed, gradually narrowing the envelope of disease detection, increasing the service station of other disease surveillance functions. Thus, the cost of testing is continuously reduced and economic pressure is released.

## An Additional Common Claim for Enhanced Disease Detection in Low- and Middle-Income Areas

Maintaining a strong database on disease surveillance and building a professional public health team to safeguard the health rights of a broader population and preserve an otherwise fragile economic environment has been a rather genuine need in low- and middle-income areas [25]. This will require a long-term joint effort by several committed and highly professional scholars in epidemiology, accounting, social sciences, and other related fields, together with low- and middle-income areas [26]. Despite the methodical efforts of disease detection and prevention in low- and middle-income areas, they are still passively waiting and receiving policy guidelines or emulation from higher levels. Much of the collection, organization, storage, and analysis of self-reported data is based on templates from other developed regions, and the cost of the vicious cycle of such a model can be devastating to some LMICs [27].

## Conclusions

Here we describe an integrated and comprehensive approach that covers structural, social, and personal strategies to optimize the epidemic surveillance system in low- and middle-income areas. This model can improve the local epidemic detection efficiency, ensure the health care needs of more people, reduce the public health costs in low- and middle-income areas in a coordinated manner, and ensure and strengthen local public health security sustainably. Although there are certain differences in the policies and environment of disease detection in each region, our framework is more intuitive and easier to understand. We hope to provide clear behavior paths and tracking paths for managers, public health workers, and participants in other collaborative events in low- and middle-income areas, and improve the possibility of all measures that can maintain local public health security.

## Abbreviations

LMIC: low- and middle-income country
NPI: nonpharmacological intervention
